# In Situ Diagnosis of Industrial Motors by Using Vision-Based Smart Sensing Technology

**DOI:** 10.3390/s19245340

**Published:** 2019-12-04

**Authors:** Ching-Yuan Chang, En-Chieh Chang, Chi-Wen Huang

**Affiliations:** Department of Mechanical Engineering, National Taipei University of Technology, Taipei 10608, Taiwan; njaycheng@gmail.com (E.-C.C.); sceddie15@gmail.com (C.-W.H.)

**Keywords:** digital image processing, early warning service, frequency monitoring, industrial motor, machine learning, non-contact inspection, preventative maintenance, support vector machine, vibration monitoring system

## Abstract

This study uses machine vision, feature extraction, and support vector machine (SVM) to compose a vibration monitoring system (VMS) for an in situ evaluation of the performance of industrial motors. The vision-based system respectively offers a spatial and temporal resolution of 1.4 µm and 16.6 ms after the image calibration and the benchmark of a laser displacement sensor (LDS). The embedded program of machine vision has used zero-mean normalized correlation (ZNCC) and peak finding (PF) for tracking the registered characteristics on the object surface. The calibrated VMS provides time–displacement curves related to both horizontal and vertical directions, promising remote inspections of selected points without attaching additional markers or sensors. The experimental setup of the VMS is cost-effective and uncomplicated, supporting universal combinations between the imaging system and computational devices. The procedures of the proposed scheme are (1) setting up a digital camera, (2) calibrating the imaging system, (3) retrieving the data of image streaming, (4) executing the ZNCC criteria, and providing the time–displacement results of selected points. The experiment setup of the proposed VMS is straightforward and can cooperate with surveillances in industrial environments. The embedded program upgrades the functionality of the camera system from the events monitoring to remote measurement without the additional cost of attaching sensors on motors or targets. Edge nodes equipped with the image-tracking program serve as the physical layer and upload the extracted features to a cloud server via the wireless sensor network (WSN). The VMS can provide customized services under the architecture of the cyber–physical system (CPS), and this research offers an early warning alarm of the mechanical system before unexpected downtime. Based on the smart sensing technology, the in situ diagnosis of industrial motors given from the VMS enables preventative maintenance and contributes to the precision measurement of intelligent automation.

## 1. Introduction

The monitoring of vibrations in industrial motors is a proven method for preventative maintenance aimed at reducing downtime [1]. Fault diagnosis depends on vibration detection using a variety of sensors [2], such as laser displacement sensors and fiber Bragg gratings to measure displacement [3,4], accelerometers to inspect acceleration [5], and radio frequency identification systems to record cycle times [6]. In many instances, the sensing point must be prearranged, since the expanding number of sensors raises the cost of mechanical systems. [7]. Laser sensors provide a non-contact approach to the measurement of dynamic signals [8]. They provide high resolution in the time domain (from ms to µs) as well as in the spatial domain (from µm scales to nm scales) [9]. Laser sensors provide either (a) high resolution with a narrow sensing range or (b) low resolution with a wide sensing region. Laser modules comprising multiple heads can help to prove biaxial measurements of displacement; however, the cost of such implementations is directly proportional to the number of heads. In comparison, accelerometers, strain gauges, and laser displacement sensors (with wires) are commercial sensors and are standard devices in the applications of instrumentation and measurement. The collection of electronic wires is experience-based works since some mechanical systems provide only limited space for those wired sensors. Electromagnetic noise also undermines the signal-to-noise ratio of those electronic devices. In addition, the increment of sensing points means raising the cost. Signal processing and artificial intelligence can enhance the numerical accuracy of the experimental results, but the computational cost may take additional processing units or edge nodes. Digital image correlation (DIC) is an optical metrology in which only one camera is used to measure biaxial displacement [10,11]. Each pixel in two-dimensional DIC (2D-DIC) serves as an independent sensor, and the core cross-correlation algorithm involves the tracking of patterns registered within an image stack [12,13]. Researchers have developed a variety of 2D-DIC algorithms to obtain high-precision measurements of systemic vibration and mechanical strain [14]. The raw data of 2D-DIC include the biaxial displacement of u(i,j) and v(i,j), in which *i* and *j* represent the indexes of the pixels at a specific frame. In addition, DIC technology can cooperate with X-ray computed tomography (CT) and measure the internal deformation of a concrete specimen [15]. The raw data of CT-DIC include the biaxial displacement of u(i,j,k) v(i,j,k), and w(i,j,k), in which *i*, *j*, and *k* represent the indexes of the voxel at a specific frame. This study has used the technology of 2D-DIC and applied the program of fast Fourier transform to analyze the vibrational features of sensing points. In this program, we adopted zero mean normalized cross-correlation in the development of our 2D-DIC program, which uses a numerical differentiation of a peak-finding program to provide subpixel resolution [16]. The 2D-DIC system provides optical superiority of non-contact inspection and yields a wide field of view, which is suitable for industrial measurements. The imaging system can measure the biaxial displacement of selected points at a remote distance and requires only one camera and one computer. The cyber–physical system is one of such concepts and is also one of the types of architecture for providing measured results to industrial instruments. This study verifies the experimental accuracy of the self-developed 2D-DIC program by using a laser displacement sensor, providing a quick diagnosis of structural health monitoring for the motor system. The vibration monitoring system uses support vector machine (SVM) criterion and provides service of preventative maintenance. The contribution of this study is to provide a cost-effective solution for industrial employments. 

Specifically, we developed a system to enable the real-time monitoring of vibration characteristics of industrial motors. Measurement results obtained using the proposed 2D-DIC systems are in strong agreement with those obtained using laser sensors. Rather than developing a closed system based on commercial software, we adopted the architecture of cyber–physical systems, which includes a physical layer, an Internet layer, and a cyber layer [17,18]. The proposed system makes it possible to use the Internet to monitor machinery in real time, thereby ensuring the immediate detection of impact loading and abnormal operations [19]. The 2D-DIC system enables the measurement of biaxial displacement at a selected point, thereby providing for trajectory in the *x-y* domain. Frequency spectra obtained using the fast Fourier transform (FFT) and short time Fourier transform (STFT) are used to illustrate the driving frequency of the industrial motor, which is fed back to a numerical controller for modulation. Edge computation is used to extract representative features from the measured displacements [20], thereby making it possible to alert the operator of abnormal operation conditions [21]. An edge node uploads the measured frequencies to the Internet via secure file transfer protocol, where it is stored in a cloud server with time stamps to enable the creation of health monitoring reports. A support vector machine is used to assist in the diagnosis of signal features extracted from the motors. After training, the VMS can also be configured to send alerts and assist in preventative maintenance. The proposed 2D-DIC system was implemented within the wireless sensor network of an existing factory. Instead of using the commercial program and open code, this study uses self-developed codes modules of 2D-DIC, SVM, and Internet service. Readers who are familiar with the DIC program can follow the proposed flowchart and modify their codes for providing customized services. The core criterion of digital image correlation is to search for predefined patterns in sequential images. Zero mean normalized cross-correlation and peak finding have demonstrated high resolution for remote inspection [22,23]. The quantitative comparisons in the temporal domain and frequency domain have shown the experimental accuracy of the self-developed vibration monitoring system (VMS) system. Support vector machine is one of the widespread criterions and can handle the DIC results quickly and efficiently. Users can choose their favorite criterion and give the same contribution of vibration monitoring. Frequency features extracted from the mechanical system as it progresses from a transient state to a steady state could prove particularly valuable in detecting early indications of motor wear. The use of image streaming also makes it possible to conduct experiments over durations ranging from seconds to days. A high signal-to-noise-ratio in the measurements of displacement helped to ensure a high degree of accuracy in fault classification.

## 2. Experiment Setup and Mathematical Model of Vibration Monitoring System

**Figure 1:**
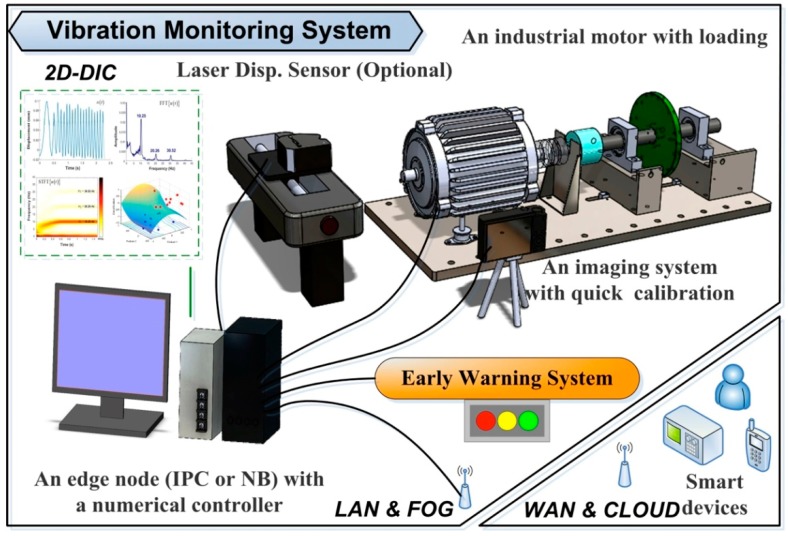
Experiment setup of proposed vibration monitoring system (VMS), which includes vision-based (i.e., non-contact) metrology as well as an early warning service to facilitate preventative maintenance.

This study developed a VMS that includes (i) an industrial motor with a numerical controller, (ii) an edge/fog computer with an imaging system, (iii) the proposed 2D-DIC program and peak-finding subroutine, (iv) a local-area network (LAN) and wide-area network (WAN), and (v) a cloud server. Figure 1 presents a schematic illustration of the VMS, showing how the industrial computer communicates with the numerical controller via a LAN connection. A laser displacement sensor was used to provide empirical results by which to verify the displacement measurements obtained using the 2D-DIC system. The VMS contains (i) a physical layer for 2D-DIC metrology, (ii) a cyber layer comprising a LAN and a WAN, and (iii) an application layer to provide preventative maintenance. The early warning system (EWS) included in the proposed 2D-DIC is not a standalone system. Rather, it is customizable to the specific requirements of the machinery and factory in which it is implemented. The proposed system is fully compliant with commercial digital cameras and industrial surveillance systems. Vectorization [16] is also used to enhance the efficiency of numerical calculation and ease the computational load on the edge node. 

In the event of an unexpected fault, the VMS alerts the operator and sends a message to the cloud server and manager. The objective is to facilitate the implementation of preventative maintenance in order to minimize downtime in the production chain. We have provided raw data of time displacement obtained from the DIC program without numerical filter. An edge computer extracts features from the spectra denoting the working status of the target motor. The primary feature is the driving frequency of the mechanical system. Other minor traits are also important and may come from the potential faults of the observed target. The computer uploads a data log of the characteristic features to the cloud server. The three layers of the VMS in Figure 1 include (i) 2D-DIC, (ii) LAN and fog/edge, and (iii) WAN and cloud. The programmability of the VMS provides flexibility in the selection of CPS architecture. 

**Figure 2:**
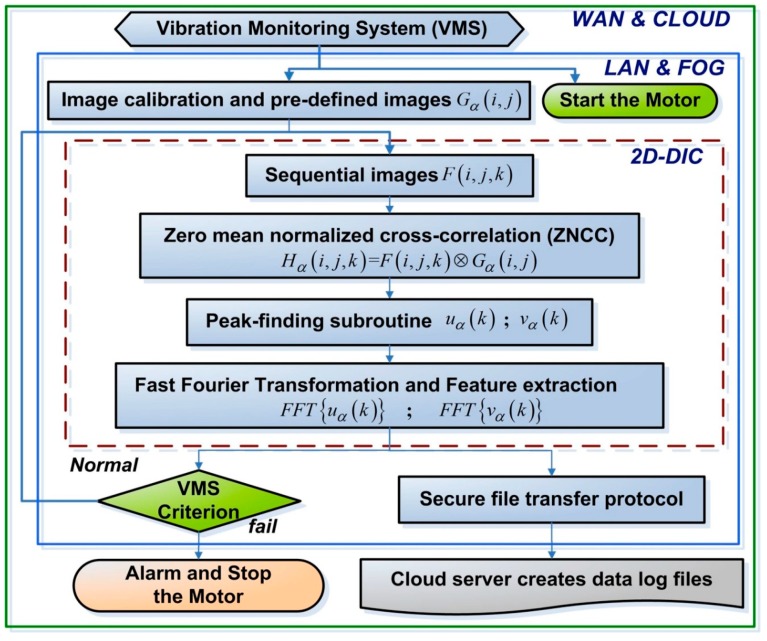
Flow chart of proposed VMS with early warning scheme (to alert users and/or halt the motor when it exceeds operational thresholds), including three layers: (i) two-dimensional DIC (2D-DIC), (ii) local-area network (LAN) and fog, and (iii) wide-area network (WAN) and cloud.

Figure 2 presents a flow chart of the proposed VMS, where *i* and *j* respectively refer to the pixel location along rows and columns; *k* is the number of selected images; and *α* is the number of selected patterns. The numerical controller governs the driving frequency of the motor, and the imaging system records sequential images F(i,j, k) and forwards them to the edge/fog computer. By tracking the patterns $G_{\alpha }\left(i,j,\;k\right)$ registered within the sequential images, the 2D-DIC program (embedded in the edge/fog computer) retrieves operating results $H_{\alpha }\left(i,j,\;k\right)$. The peak-finding program provides a theoretical subpixel resolution of 0.01 pixels, wherein horizontal displacement is indicated by $u_{\alpha }\left(k\right)$, whereas vertical displacement is indicated by $v_{\alpha }\left(k\right)$. The VMS system uses the fast Fourier transforms $FFT\left\{u_{\alpha }\left(k\right)\right\}$ and $FFT\left\{v_{\alpha }\left(k\right)\right\}$ to obtain spectra of vibrations associated with horizontal and vertical displacement. 

In this study, we conducted four experiments to verify the functionality of the proposed VMS: (i) constant frequency, (ii) acceleration under unbalanced loading, (iii) acceleration under the effects of external perturbation, and (iv) a ramp function under the effects of external perturbation. The first experiment assesses the stability of the VMS and 2D-DIC, wherein the FFT and STFT spectra were used to determine the working status of the target motor. The second experiment presents the experimental accuracy based on the benchmark of the 2D-DIC and LDS, wherein the motor was driven from an inactive state until it reached a stable state. The third experiment illustrates the performance of the motor under the effects of perturbation, and demonstrates the efficacy of the VMS probes in detecting noise and sending alerts based on analysis obtained from the support vector machine. The fourth experiment illustrates that the fault classification of SVM is based on extracted features along the horizontal and vertical directions, and enhances the accuracy of the support vector machine. This study composed the DIC and CPS system for solving the real problems of the mechanical system, providing a reliable and economical solution for industrial measurement.

**Figure 3:**
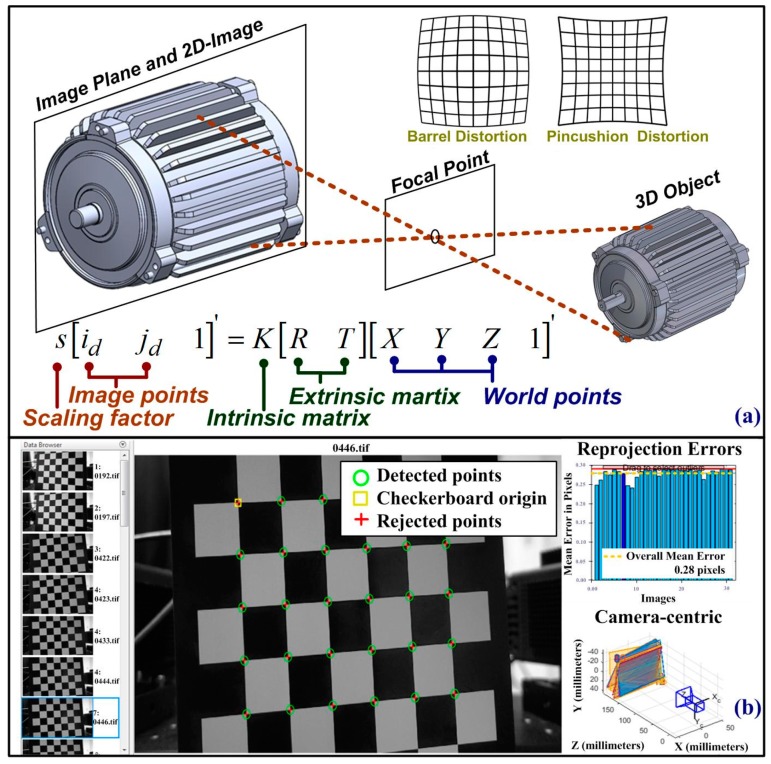
Optical system of proposed VMS for non-contact inspection: (**a**) projection matrix of single-camera system and (**b**) device calibration using checkerboard image.

Figure 3a presents a schematic illustration of the imaging system, and the following equations are used to describe the projection matrix [24]:

(1) $$s\left[\begin{array}{c} i_{d}\\ j_{d}\\ 1 \end{array}\right]=\left[\begin{array}{ccc} f_{x} & 0 & c_{x}\\ 0 & f_{y} & c_{y}\\ 0 & 0 & 1 \end{array}\right]\left[\begin{array}{cccc} r_{11} & r_{12} & r_{13} & t_{1}\\ r_{21} & r_{22} & r_{23} & t_{2}\\ r_{31} & r_{32} & r_{33} & t_{3} \end{array}\right]\left[\begin{array}{c} X\\ Y\\ Z\\ 1 \end{array}\right]=K\left[\begin{array}{cc} R & T \end{array}\right]{\left[\begin{array}{cccc} X & Y & Z & 1 \end{array}\right]}^{\prime }$$

where (X,Y,Z) refers to the 3D location of selected measured points; and (x,y,z) are used to specify the location of the measured points in three dimensions. $\left(i_{d},j_{d}\right)$ indicates the location of pixels in an image distorted along the horizontal and vertical directions. *R* is a rotation matrix containing all parameters of $r_{11},r_{12},\ldots ,r_{33}$; *T* is a translation matrix including $t_{1},t_{2},t_{3}$; the extrinsic matrix is a 4 × 3 matrix constructed using *R* and *T*. $\left(c_{x},c_{v}\right)$ is a principal point that is usually at the image center; $\left(f_{x},f_{v}\right)$ are the focal lengths expressed in pixel units, giving $\left(F/p_{x},\;F/p_{v}\right)$; F is the focal length in world units, typically expressed in millimeters. $\left(p_{x},\;p_{v}\right)$ is the size of the pixel in world units. The subscript of *x* and *y* denotes the direction of the horizontal and vertical direction, respectively. *K* is an intrinsic matrix; *s* is a scaling factor for the 2D imaging system. Image correlation between the distorted image and the undistorted image along horizontal and vertical projection gives [24]

(2) $$i_{d}=i_{d}^{r}+i_{d}^{t}=\left\{i+\left[2p_{1}ij+p_{2}\left(r^{2}+2i^{2}\right)\right]\right\}+\left\{i\left(k_{1}r^{2}+k_{2}r^{4}+k_{3}r^{6}\right)\right\}$$

(3) $$j_{d}=j_{d}^{r}+j_{d}^{t}=\left\{j+\left[p_{1}\left(r^{2}+2j^{2}\right)+2p_{2}ij\right]\right\}+\left\{j\left(k_{1}r^{2}+k_{2}r^{4}+k_{3}r^{6}\right)\right\}$$

where $r=\sqrt{i^{2}+j^{2}}$. In these formulas, *i* and *j* indicate locations in an undistorted image; $i_{d}$, $j_{d}$ are the pixel locations of the distorted image; and superscripts *r* and *t* denote radial and tangential distortion in the 2D imaging system. Figure 3b presents the results of image calibration performed using a checkerboard pattern, and this work uses 12 images with random positions to compensate for image distortion. The image calibration subroutine computes the coefficient of $\left[\begin{array}{ccccc} p_{1} & p_{2} & k_{1} & k_{2} & k_{3} \end{array}\right]$ in order to compensate for radial distortion and tangential distortion in the 2D imaging system. Figure 3a has presented the schematic picture of the image calibration, and Equations (1)–(3) have shown the mathematical model of numerical computations. Figure 3b shows that the imaging system recorded 12 frames of a checkerboard placed at a random position, in which green circles indicate intersection points between black and white blocks. The program of image calibration computes the transform matrix between the detect points in the projected image plane and the corresponding coordinates in the real world. The MATLAB program provides camera parameters of an intrinsic matrix, an extrinsic matrix, and the lens distortion parameters of a camera.

The motor performs 3D vibrations and the systematic vibration is decoupled into three independent functions, giving

(4) S(x,y,z,t)=U(x,y,z,t)+V(x,y,z,t)+W(x,y,z,t)

where U(x,y,z,t), V(x,y,z,t), and W(x,y,z,t) respectively refer to decoupled deformation along the horizontal, vertical, and depth directions. The self-developed program follows the architecture of object orientation and preserves high flexibility and originality for practical applications. The corresponding variables in the spatial domain are *x*, *y*, and *z*.

The single camera system used in the proposed VMS measures only the in-plane vibration and therefore cannot represent *z* or W(x,y,z,t). Thus, we use a downscaled 3D model to represent in-plane deformation based on U(x,y,t), V(x,y,t), as follows:

(5) $$S^{*}\left(x,y,t\right)=u\left(x,y,t\right)+v\left(x,y,t\right)\;=U\left(x,y,t\right)\Delta W_{x}\left(x,y,t\right)+V\left(x,y,t\right)\Delta W_{y}\left(x,y,t\right)$$

where $\Delta W_{x}\left(x,y,t\right)$ and $\Delta W_{y}\left(x,y,t\right)$ are the scaling factors derived from out-of-plane deformation, which are used to modulate the 2D measurement results indicating horizontal and vertical displacement, respectively. u(x,y,t) and v(x,y,t) represent the systemic vibrations in the 2D formation. We used the zero-mean normalized correlation (ZNCC) and peak finding (PF) for tracking the registered characteristics on the object surface, giving [16]

(6) $$H_{\alpha }\left(i,j;\;k\right)=\frac{\sum_{l=-L}^{L}\sum_{m=-M}^{M}\left[F\left(i+l,j+m;\;k\right)-\bar{F}\left(i,j;\;k\right)\right]\left[G_{\alpha }\left(l,m\right)-{\bar{G}}_{\alpha }\right]}{\sqrt{\sum_{l=-L}^{L}\sum_{m=-M}^{M}{\left[F\left(i+l,j+m;\;k\right)-\bar{F}\left(i,j;\;k\right)\right]}^{2}}\sqrt{\sum_{l=-L}^{L}\sum_{m=-M}^{M}{\left[G_{\alpha }\left(l,m\right)-{\bar{G}}_{\alpha }\right]}^{2}}}$$

(7) $$\bar{F}\left(i,j;\;k\right)=\frac{1}{\left(2L+1\right)\left(2M+1\right)}\sum_{l=-L}^{L}\sum_{m=-M}^{M}F\left(i+l,j+m;\;k\right)$$

(8) $${\bar{G}}_{\alpha }=\frac{1}{\left(2L+1\right)\left(2M+1\right)}\sum_{l=-L}^{L}\sum_{m=-M}^{M}G_{\alpha }\left(l,m\right)$$

where the F(i,j; k) denotes an image stack and k is the index of frame. $G_{\alpha }\left(l,m\right)$ are cropped images from the image stack, and the subscript α is the index of the registered pattern. $H_{\alpha }\left(i,j;\;k\right)$ is the computed coefficient of ZNCC, which ranges from 0 to 1. The location of the peak indicates the most matched point of $G_{\alpha }\left(l,m\right)$ and F(i,j; k), giving

(9) $$\left[u_{\alpha }^{P}\left(k\right),v_{\alpha }^{P}\left(k\right)\right]=\max\left(H\left(i,j;k\right)\right),\;\mathrm{where}\;k=2,\ldots ,K.$$

The peak location has been decoupled into $u_{\alpha }^{P}\left(k\right)$ and $v_{\alpha }^{P}\left(k\right)$ in the row and column direction, respectively. The superscript of P denotes the pixel resolution instead of subpixel resolution by using numerical differentiation. Following calibration, the 2D-DIC program provides quantitative results for $u_{\alpha }\left(k\right)$ and $v_{\alpha }\left(k\right)$. Image calibration based on the checkerboard image provides a projection matrix by which to correct the distorted pixel $\left(i_{d},j_{d}\right)$ into undistorted locations (*i*, *j*). Timestamps of the recorded images permit the creation of a 1D array, wherein time *t* is converted into a frame number represented as *k*. The FFT is used to extract features pertaining to systemic vibration as follows:

(10) $${\tilde{u}}_{\alpha }\left(\omega \right)\;=\sum_{K=0}^{K-1}u_{\alpha }\left(k\right)e^{-i2\pi k\omega /K}$$

(11) $${\tilde{v}}_{\alpha }\left(\omega \right)\;=\sum_{K=0}^{K-1}v_{\alpha }\left(k\right)e^{-i2\pi k\omega /K}$$

where *K* is the total number of recorded images and *ω* is the frequency variable. We note that *α* is the number of selected patterns instead of the variable of frequency. Out-of-plane deformation undermines the numerical resolution of 2D-DIC in the time domain; however, the signal-to-noise ratio (SNR) in the frequency domain is high enough to characterize the status of the motor in terms of normal operations and failure. For a given point, the frequency spectrum in a clip interval gives ${\tilde{u}}_{\alpha }\left(\omega \right)$, where *α* is a free index denoting the number of the clipped signals. F{} denotes the operation of feature extraction, and the extracted feature performs a vector of ${\vec{\psi }}_{\alpha }$, giving

(12) $${\vec{\psi }}_{\alpha }=F\left\{{\tilde{u}}_{\alpha }\left(\omega \right)\right\}={\left[\begin{array}{ccccc} \psi_{1} & \psi_{2} & \psi_{3} & \cdots & \psi_{n} \end{array}\right]}_{\alpha }$$

where $\psi_{1}$, $\psi_{2}$, $\psi_{3}$ to $\psi_{n}$ are extracted features. Measured data $\left({\vec{\psi }}_{\alpha },\phi_{\alpha }\right)$ are used to construct a hyperplane for the support vector machine (in the VMS), which is used to characterize the health status $\phi_{\alpha }=1$ and detect failure status $\phi_{\alpha }=-1$. The normal vector of the hyperplane $\vec{w}$ gives us the following:

(13) $$\vec{w}\cdot {\vec{\psi }}_{\alpha }=b$$

where *b* is the distance between selected points and the hyperplane. The feature extraction used in this study is based on FFT and STFT and can cooperate with wavelet transformation for different applications.

**Figure 4:**
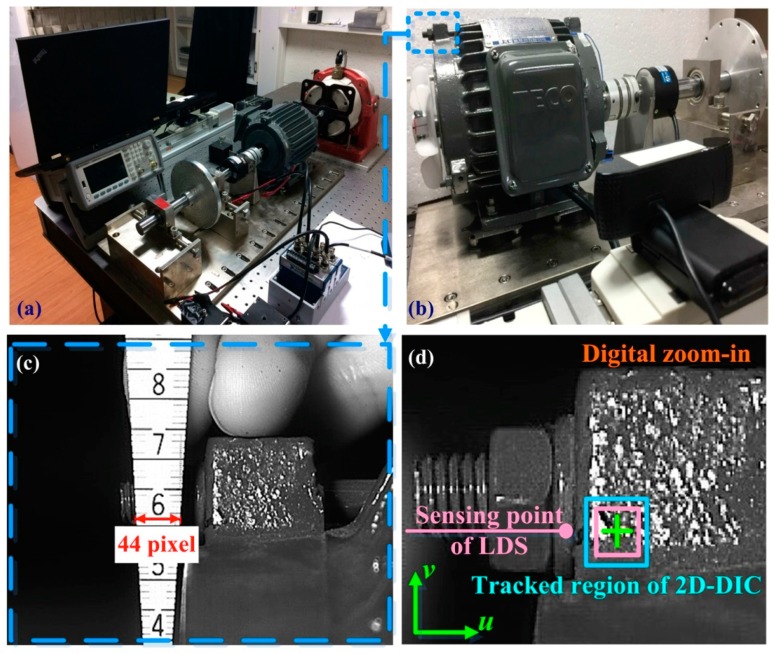
(**a**) Experimental setup used to evaluate proposed VMS system; (**b**) single USB camera for in-plane measurement of horizontal and vertical displacement; (**c**) 2D-DIC program designed to track nature features on surface of target motor; (**d**) region tracked by 2D-DIC (note that this area was close to the sensing point used by the laser displacement sensor).

Figure 4a presents the setup of a dynamic balance experiment in which the VMS was applied to a motor (AEHF 1HP, TECO) driving a balanced wheel. The maximum frequency of the motor was 1730 RPM and the maximum power was 746 Watt (1 horsepower). External vibrations were introduced using an external shaker to verify the functionality of the early warning system. The system computer was connected to a numerical controller via an Ethernet connection. Figure 4b presents the imaging system and USB camera used in the experiment (resolution = 1920 × 1080; and average frame rate = 60 frames per second) (Brio, Logitech, United States). In the 2D-DIC system, the left-top corner is the region of interest (ROI). Figure 4c presents the gauge factor between the actual length and the image pixels. The spatial resolution in these experiments was 1.4 µm, which was based on the results of image calibration and the specifications of the peak-finding subroutine. Following image calibration, the 2D-DIC system provides quantitative results of biaxial displacement. The origin of the 2D-DIC system was located in the bottom-left corner of the recorded image. Figure 4d presents the region tracked by the 2D-DIC system, which was close to the sensing point used by the laser displacement sensor (LK-H050, Keyence, Japan). The resolution of the laser displacement sensor was 0.025 µm. The 2D-DIC and LDS systems both provide quantitative results, although the 2D-DIC provides biaxial displacement, whereas the LDS provides measurements of higher precision. However, the fact that the proposed VMS requires only one camera and a relatively simple computer system makes it a cost-saving solution for a wide range of industrial applications.

## 3. Measured Results of Biaxial Displacement and Frequency Spectra

**Figure 5:**
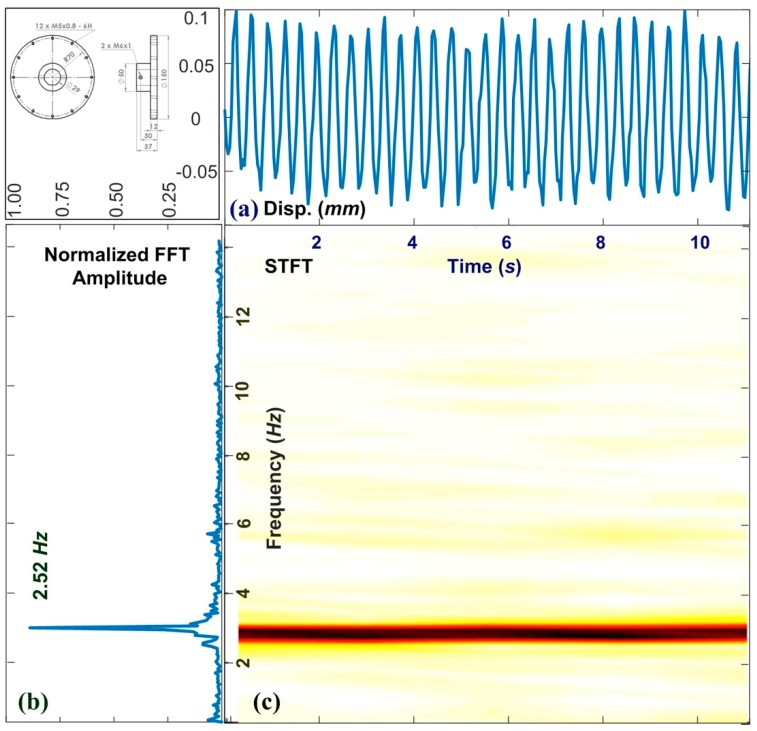
2D-DIC frequency analysis of motor operations at stable speed of 150 RPM: (**a**) time–displacement curve; (**b**) fast Fourier transform; and (**c**) short-time Fourier transformation. The major feature appeared at 2.52 Hz.

Figure 5 presents the experiment results obtained from a motor exhibiting vibration at a constant frequency. Figure 5a–c respectively present the measured results of horizontal displacement, the FFT spectrum, and STFT spectrum. In this experiment, the revolution of the motor was 150 RPM and the measured frequency shown in Figure 5c was 2.52 Hz. The discrepancy may come from the minimum resolution of the numerical controller. The average frame rate of the USB camera was 60 frames per second, which should be sufficient to measure vibrations in the system. The high degree of linearity in the frequency measurements verifies the stability of the 2D-DIC program. In a steady state, the major feature of $\psi_{1}$ is clearly defined; however, the minor features ($\psi_{2}$ and $\psi_{3}$) are somewhat unclear. We note that the *y*-axis of FFT in amplitude (linear scale) can provide more obvious traits than those in dB (log scale). This type of vision-based metrology reduces the time and cost of implementation. This, in turn, makes it possible to increase the number of sensing nodes used to monitor the health of industrial devices over extended durations. We present a clipped signal from image streaming and use a Hanning window with 1024 seconds. Figure 5a,b shows the zoom-in results of time-domain and the frequency spectrum within the window. Figure 5c demonstrates the stability of the boundary conditions and operating parameters. The measured frequency is 2.52 Hz instead of 2.50 Hz. The discrepancy is 0.8% and demonstrates the accuracy of the VMS. The proposed VMS and 2D-DIC can identify the difference between the numerical parameters given by the digital controller and the real performance shown in the mechanical system. These results show that the proposed VMS provides a high degree of stability and high accuracy, while enabling the monitoring of motors over extended durations. 

**Figure 6:**
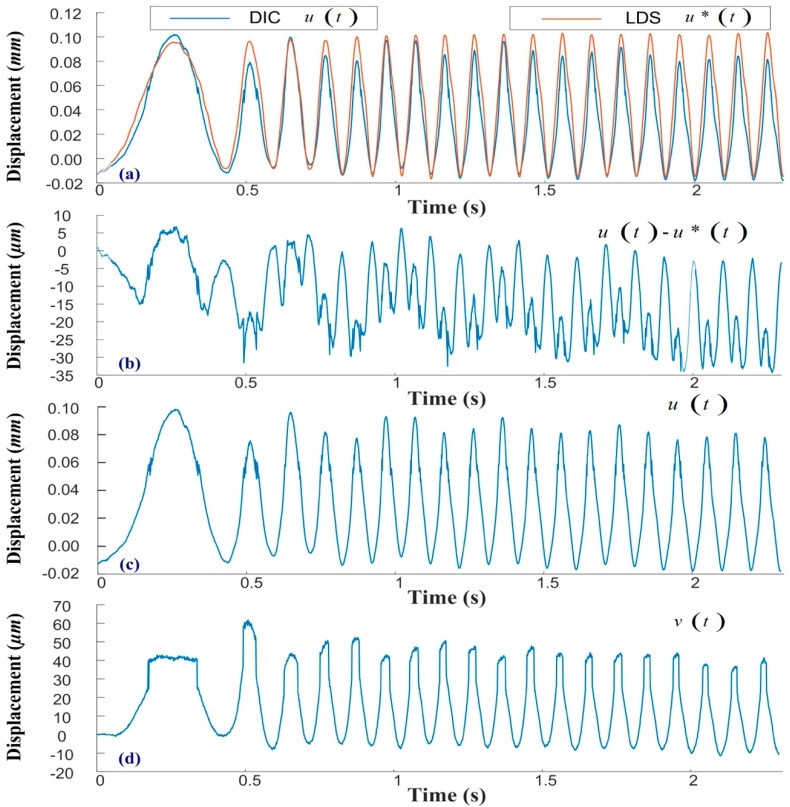
Quantitative comparison of results obtained using 2D-DIC and laser displacement sensor (LDS): (**a**) quantitative comparison of metrologies; (**b**) maximum discrepancy between metrologies (less than 62 µm); (**c**) horizontal displacement derived using the 2D-DIC system; (**d**) vertical displacement derived using the 2D-DIC system.

Figure 6 presents the experiment results of acceleration from a static state to 600 RPM (the time used in acceleration is 0.5 seconds) under an unbalanced load, and the quantitative comparison in the time domain. Figure 6a shows the horizontal displacements, as obtained from 2D-DIC (u(t)) and LDS ($u^{*}\left(t\right)$). The consistency between these two sets of results verifies the feasibility of the proposed vision-based VMS. Note that the 2D-DIC system provides $u\left(t\right)\;=U\left(t\right)\Delta W_{x}\left(t\right)$ which coupled in the time domain, where $\Delta W_{x}\left(t\right)$ is a scaling factor that induces uncertainty in experimental measurement. Figure 6b illustrates the difference between u(t) and $u^{*}\left(t\right)$, which reached 62 µm and may be attributable to $\Delta W_{x}\left(t\right)$. Out-of-plane deformation was shown to undermine accuracy in the time domain; therefore, the VMS program was set to extract features from the frequency domain. Figure 6c,d respectively present the horizontal and vertical displacements obtained from the 2D-DIC system. The maximum u(t) value was 0.97 mm and the maximum v(t) value was 62 µm, the ratio of which was 15.64. This is a clear demonstration of the high resolution and extended working distance made possible by the proposed 2D-DIC system.

**Figure 7:**
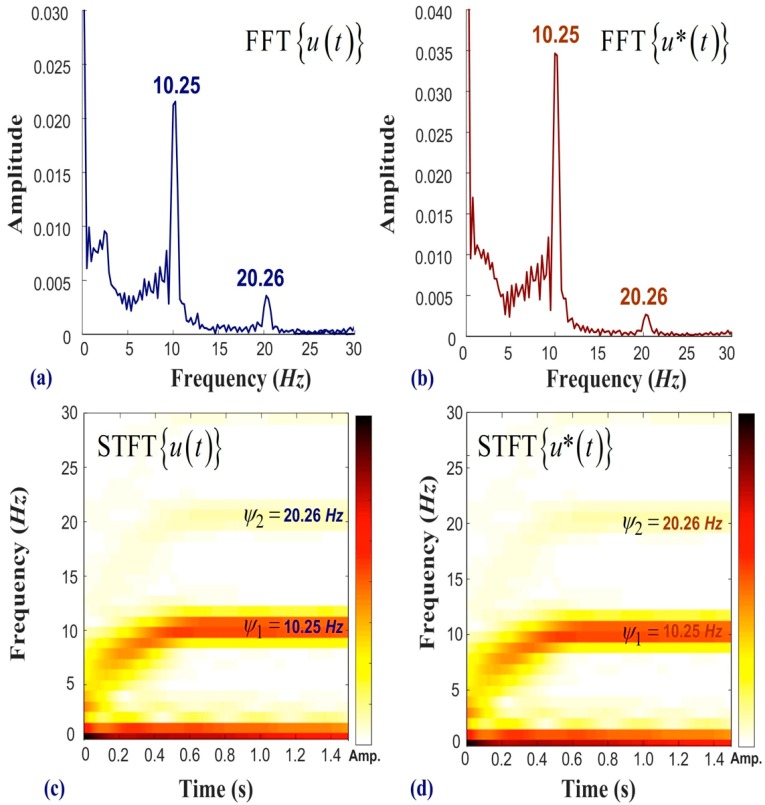
Analysis of frequency results obtained using 2D-DIC and LDS with motor accelerated from static status to 600 RPM: (**a**) frequency spectra of FFT{u(t)}; (**b**) frequency spectra of $FFT\left\{u^{*}\left(t\right)\right\}$; (**c**) short-time Fourier transform of u(t); and (**d**) short-time Fourier transform of u*(t).

Figure 7 presents the experiment results of acceleration under an unbalanced load using a measured signal in the frequency domain. Figure 7a,b respectively present the frequency spectra of u(t) and $u^{*}\left(t\right)$. The results obtained using 2D-DIC and LDS are nearly identical, thereby demonstrating the accuracy of the proposed system. In this experiment, the numerical controller designated a motor speed of 600 RPM corresponding to a frequency of 10 Hz; however, the measured result was 10.25 Hz. The industrial motor features open loop control and there may exist a slight discrepancy between the asked frequency and the measured frequency. In this case, the major feature is the working frequency of the motor (i.e., 10.25 Hz). The minor feature at 20.26 Hz also provides information for the VMS program. Figure 7c,d respectively present the u(t) and $u^{*}\left(t\right)$ results obtained using STFT. The observed increase in frequency indicates acceleration of the motor, which reached a steady state at 0.45 s. The results of FFT and STFT verify that the influence from scaling factor $\Delta W_{x}\left(t\right)$ was acceptable, and the extraction of features from the first three frequencies proved workable for the early warning alarm. The extracted features ($\psi_{1}$, $\psi_{2}$ and $\psi_{3}$) respectively correspond to the first, second, and third peaks in the frequency spectrum.

**Figure 8:**
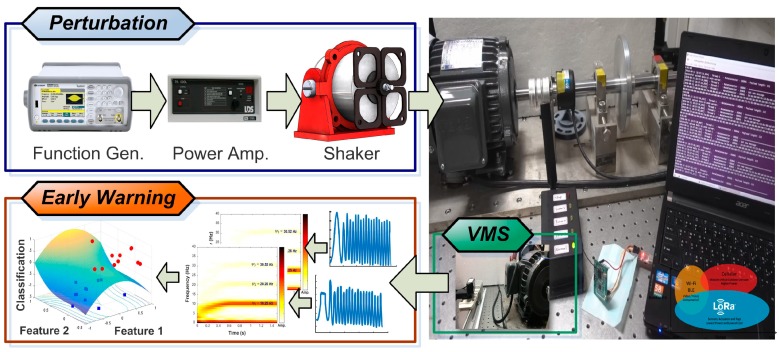
Shaker device used to introduce perturbations into the operation of a motor and VMS early response system designed to forward alerts to the cloud-based server and send messages to operators. VMS facilitates the implementation of preventative maintenance according to the protocol for that operating environment.

Figure 8 presents the experimental setup of a shaker-equipped motor (LDS V406, Bruel & Kjaer), the proposed VMS, and the early warning system. A function generator (33120A, keysight technology, United States) was used to generate a predefined waveform to produce external vibrations that would perturb the motor during acceleration. An optical table (Model 78-236-12R, TMC, United States) and uninterruptable power system with ground (SUA3000RM2U, APC, United States) provides the VMS system a stable environment of low noise for the mechanical system and electronic devices. The primary direction of the perturbations was along the horizontal axis. The VMS consider vibrational features from both the horizontal and vertical directions. Frequency spectra based on FFT and STFT were used to identify the traits of the motor, and machine learning was used to classify the faults based on a hyperplane. The edge node uploads the following extracted features: (i) peak value of measured frequency, (ii) full-width at half-maximum of the peak frequencies, and (iii) the difference between the value of $\psi_{1}$ and $\psi_{2}$. Figure 8 also presents a schematic illustration showing the hyperplane constructed using the support vector machine. The proposed VMS metrology makes it possible to obtain precision measurements in the horizontal and vertical directions in real time from a remote location. The system can be implemented using a commercial USB camera, wherein each pixel serves as an independent sensor. The DIC program tracks a predefined pattern within sequential images based on zero-mean normalized cross-correlation. Numerical differentiation is used in a peak-finding subroutine providing subpixel resolution. We observed a high degree of consistency between the results of the proposed DIC system and those obtained using laser displacement sensors, as given in Figure 6a. The operating health of the motors is monitored in situ through the use of short-time Fourier transform diagrams indicating fluctuations in frequency and the occurrence of abnormal behavior. Implementation of the proposed imaging system requires only basic image calibration and axis calibration. This study has used vision-based technology to inspect mechanical vibrations in industrial motors and provided a cost-effective framework for cyber–physical systems aimed at enhancing the stability of production chains. We use MATLAB 2018b and the frameworks of object orientation to compose the VMS program, which properly integrated the functions of ZNCC, PF, SVM, graphic user interface, and Internet protocol. The DIC metrology gives biaxial displacement to the real-time observation of systematic vibration, providing high SNR for analyzing the biaxial vibration of the industrial motor. Developers can use the apparent features to downscale the number of training layers and to save the time cost used in the SVM computation. Verified users can use smart devices (cellphones, tablets, laptops) to obtain the in situ diagnosis of the observed motors.

**Figure 9:**
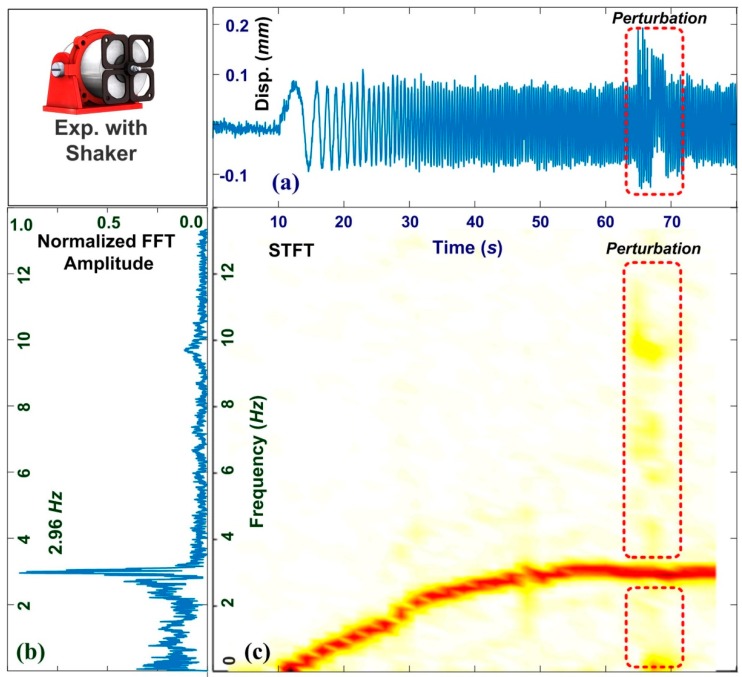
Feature analyses of signals obtained from motor during acceleration under effects of external perturbation: signals in the (**a**) time domain, (**b**) frequency domain, and (**c**) corresponding STFT spectrum. The motor was accelerated from static state to 176 RPM and external perturbations were introduced at 63.5 seconds. Features imposed by perturbations can be clearly observed in signals in the time domain as well as in the STFT spectrum. These unexpected features would be sufficient to trigger alerts by the VMS program.

Figure 9 presents feature analyses of motor acceleration under the effects of perturbation. Figure 9a shows the signal obtained in the time domain, in which the amplitude of the vibrations ranged from 0.12 to −0.08 mm before the onset of perturbation at 63.5 s. External noise was shown to impose an additional displacement of 0.08 mm. Figure 9b shows the frequency spectrum obtained using FFT, from which it was not possible to distinguish perturbations from the gross results. Figure 9c presents the STFT spectrum showing the increasing frequency of the motor. The primary line indicates the working frequency. The motor attained a stable rate of 2.96 Hz after 50 s. The perturbation at 63.5 s induced minor fluctuations, which triggered the VMS alarm. Users can observe the transient features of the industrial motor at a remote distance. The VMS provides the in situ measurement of STFT spectra. We note that the support vector machine is one of the criteria used in this study, which provides the most straightforward results. Users can select a proper camera for meeting the different requirements of structural health monitoring. They also can choose favorite criteria, and give the same contribution of vibration monitoring. The spatial resolution and temporal resolution relies on the frame rate and total pixels in one frame. The temporal resolution of the VMS is 16.6 ms and is high enough to resolve the dynamic response of the industrial motor. The VMS program can extract features from the time domain and frequency domain, enriching the dataset used in the SVM and increasing the accuracy of the early warning alarm.

**Figure 10:**
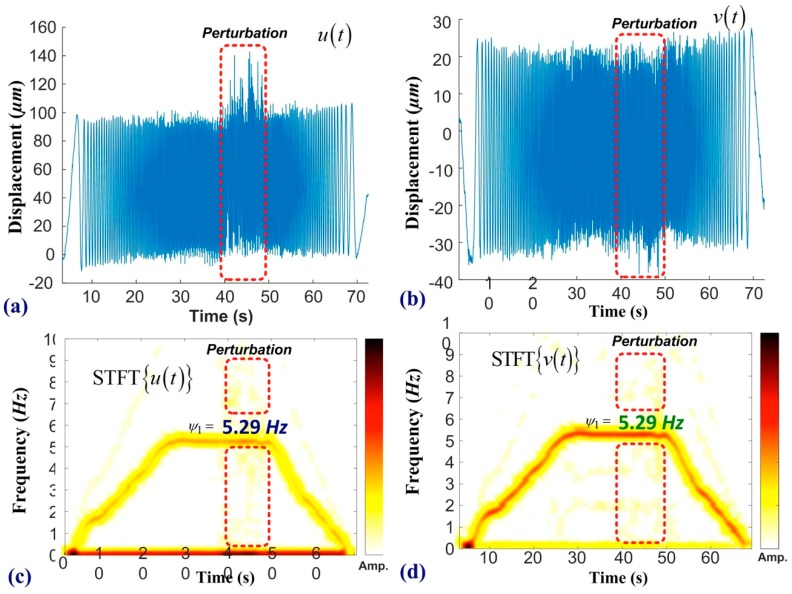
Frequency analysis of motor undergoing ramp function: (**a**) horizontal displacement and (**b**) vertical displacement. Perturbations were introduced between 40 and 50 seconds. Short-time Fourier transform (STFT) spectra along the (**c**) horizontal direction and (**d**) vertical direction reveal features pertaining to the operational state of the motor. The rectangular area indicates noise induced by the shaker.

Figure 10 presents the experiment results of the ramp function under the effects of perturbation. In this experiment, the working frequency of the motor was increased from a static state to 318 RPM and then decreased from 318 RPM back to the static state. Figure 10a,b respectively presents time-domain signals measured in the horizontal and vertical directions, with perturbations imposed between 40 and 50 s. Before the onset of noise, the average peak-to-peak amplitude of the motor was 98.3 µm in the horizontal direction and 61.2 µm in the vertical direction. Figure 10c,d respectively present STFT spectra along the horizontal and vertical directions, where the red line denotes the primary frequency of the motor. We observe slight differences between the requested value by the numerical controller (5.30 Hz) and the measured results (5.29 Hz). The VMS provides biaxial displacement of the sensing point, and Figure 10 presents that the horizontal vibration dominates the mechanical oscillation, and the primary frequency (5.29 Hz) shown in Figure 10c,d are consistent each other. This discrepancy (<0.3 Hz) may be attributed to a time delay associated with the frequency command. Precision measurements of biaxial vibration provide more comprehensive information than those given from LDS. The high SNR and obvious features help the SVM program effectively construct a hyperplane for preventative maintenance, as shown in Figure 8. The main contribution of this work is our development of an early warning system for motor malfunction based on the 2D-DIC metrology. The non-contact inspection based on machine vision can cooperate with different programs of machine learning and deep learning. The proposed VMS is a single-camera system, which features simple installation and the rapid measurement of biaxial displacement. The proposed scheme could be extended to include multiple cameras by which to measure vibrations in three dimensions, based on stereo vision. Local computers embedded with the proposed 2D-DIC program can be used to conduct remote inspections of motors through the measurement of biaxial displacement.

## 4. Discussion

In motor factories, some machines are standalone and do not have an Internet connection. The measurement based on probes requires recalibration if the specifications of the rotors change. Edge computers equipped with accelerometers have a well-developed metrology for vibration monitoring, but the increasing number of sensing points results in a rising cost. Fiber optics offers a high signal-to-noise ratio, but the price of a broadband light source and an optical spectrum analyzer is high (over USD 6000). This study constructed a vision-based metrology and provided consistent agreement with those obtained from a commercial LDS. The proposed VMS promises time-saving processes for experiment setup and offers a cost-effective selection for industrial applications. The major limitation of vision-based smart sensing technology is the illumination. A sufficient flux of light is necessary for recording clear images, and the user can select proper devices for the image preprocessing. The minor limitation of the VMS is the specification of the imaging system. The spatial resolution and time resolution depends on the pixel per frame and frames per second, respectively. The proposed VMS system provides customized services for different industrial machines, saving all the raw data at a local drive and only uploading critical features to a cloud server. Authorized users can access the production report for smart management. In Figure 4, the selection of the ROI is manual, and users can select multiple ROI for the long-term observation. They also can change the selection of the ROI during the VMS program working. Different cameras provide a different field of view, and one camera can provide multiple sensing points on an observing plane. The proposed VMS is non-contact metrology and is a cost-effective choice if compared with laser displacement sensors. The system provides a higher resolution both in the spatial domain and time domain than those of wired and low-cost sensors. Engineers can customize programs for satisfying different requirements of precision measurement and industrial applications. When the time window is 1024 s, the observation of transient response is unobvious, and there is a time delay in the inspection of structural vibration. We note that the larger the motor, the lower the working frequency. Our camera provides 60 frames per second, which is high enough to decrypt the structural vibration under 1800 RPM. The specification of the imaging system can satisfy most cases of industrial motors.

## 5. Conclusions

This paper presents a vibration monitoring system based on machine vision, edge computing, and cloud computing. The proposed VMS proved to be highly robust when applied to real-time measurement. The 2D-DIC metrology can be implemented using most commercial USB camera systems. In experiments, the displacement measurements obtained using the 2D-DIC scheme (at a spatial resolution of 1.4 µm and temporal resolution of 16.6 ms) are in good agreement with those obtained using laser displacement sensors. The enhancement of image pixels and frame rate can increase the spatial and temporal resolution of the DIC computation. A support vector machine is used to extract features from the vibration patterns derived using FFT and STFT. Experiments were conducted to assess the efficacy of the proposed scheme under four scenarios: (i) constant frequency, (ii) acceleration under unbalanced loading, (iii) acceleration under the effects of external perturbation, and (iv) a ramp function under the impact of external disturbance. A local node uploads downscaled data (including essential features) to a cloud server using the secure file transfer protocol, whereupon an early warning system sends an alert to the smart device of the operator to make them aware of unexpected variations in the operation of the motor, thereby facilitating preventative maintenance in a timely manner.
